# Barriers to evidence use for sustainability: Insights from pesticide policy and practice

**DOI:** 10.1007/s13280-022-01790-4

**Published:** 2022-11-17

**Authors:** Benjamin Hofmann, Karin Ingold, Christian Stamm, Priska Ammann, Rik I. L. Eggen, Robert Finger, Samuel Fuhrimann, Judit Lienert, Jennifer Mark, Chloe McCallum, Nicole Probst-Hensch, Ueli Reber, Lucius Tamm, Milena Wiget, Mirko S. Winkler, Lucca Zachmann, Sabine Hoffmann

**Affiliations:** 1 Department of Environmental Social Sciences, Eawag, Swiss Federal Institute of Aquatic Science and Technology, Überlandstrasse 133, 8600 Dübendorf, Switzerland; 2 Institute of Political Science, University of Bern, Fabrikstrasse 8, 3012 Bern, Switzerland; 3 Oeschger Centre for Climate Change Research, University of Bern, Hochschulstrasse 4, 3012 Bern, Switzerland; 4 Department of Environmental Chemistry, Eawag, Swiss Federal Institute of Aquatic Science and Technology, Überlandstrasse 133, 8600 Dübendorf, Switzerland; 5 Department of Epidemiology and Public Health, Swiss Tropical and Public Health Institute, Kreuzstrasse 2, 4123 Allschwil, Switzerland; 6 University of Basel, Basel, Switzerland; 7 Directorate, Eawag, Swiss Federal Institute of Aquatic Science and Technology, Überlandstrasse 133, 8600 Dübendorf, Switzerland; 8 Department of Environmental Systems Science, ETH Zürich, Zurich, Switzerland; 9 Agricultural Economics and Policy Group, ETH Zürich, Sonneggstrasse 33, 8092 Zurich, Switzerland; 10 Department of Crop Sciences, FiBL: Research Institute of Organic Agriculture, Ackerstrasse 113, 5070 Frick, Switzerland; 11 Faculty of Medicine, University of Basel, Basel, Switzerland; 12 TdLab, Department of Environmental Systems Science, ETH Zürich, Universitätstrasse 16, 8092 Zurich, Switzerland

**Keywords:** Agriculture, Evidence, Pesticides, Policy and practice, Sustainability, Transformation

## Abstract

Calls for supporting sustainability through more and better research rest on an incomplete understanding of scientific evidence use. We argue that a variety of barriers to a transformative impact of evidence arises from diverse actor motivations within different stages of evidence use. We abductively specify this variety in policy and practice arenas for three actor motivations (truth-seeking, sense-making, and utility-maximizing) and five stages (evidence production, uptake, influence on decisions, effects on sustainability outcomes, and feedback from outcome evaluations). Our interdisciplinary synthesis focuses on the sustainability challenge of reducing environmental and human health risks of agricultural pesticides. It identifies barriers resulting from (1) truth-seekers’ desire to reduce uncertainty that is complicated by evidence gaps, (2) sense-makers’ evidence needs that differ from the type of evidence available, and (3) utility-maximizers’ interests that guide strategic evidence use. We outline context-specific research–policy–practice measures to increase evidence use for sustainable transformation in pesticides and beyond.

## Introduction

To support sustainable development and biodiversity conservation, scholars have been calling for expanding sustainability science (Messerli et al. 2019), re-thinking knowledge production and use (Abson et al. 2017), promoting action-oriented knowledge (Caniglia et al. 2021), and co-producing knowledge among researchers, policymakers, and practitioners (Cvitanovic and Hobday 2018; Norström et al. 2020). Such calls build on—often implicit—assumptions about why scientific evidence succeeds or fails to generate impact. The impact may depend on scientists’ evidence supply, policymakers’ and practitioners’ evidence demand, or the matching of both (McNie 2007). However, empirical analyses of evidence use remain fragmented across disciplines and focused on selected processes and actors (Oliver and Boaz 2019). This makes it difficult to fully understand the barriers to evidence use for sustainability, which is characterized by multidimensionality, complex processes, and diverse actors. Consequently, the analytical basis for debating the future directions of sustainability research is incomplete.

We argue that the role of scientific evidence needs to be studied through actor motivations and stages of evidence use. Studying motivations within different stages helps understand the manifold barriers to evidence use for transforming socio-ecological systems toward greater sustainability. We consider that actors in the policy and practice arenas are driven by three motivations (truth-seeking, sense-making, and utility-maximizing) in five stages (evidence production, uptake, influence on decisions, effects on sustainability outcomes, and feedback from evaluating effects). Combining motivations and stages expands actor-centered work on science impacts and promises fine-grained assessments that can inform context-specific research–policy–practice strategies to increase evidence use for sustainability.

We illustrate our argument using the environmental and human health risks of agricultural pesticides as a typical sustainability problem. Pesticide risk reduction is a complex socio-ecological challenge at the food-health-environment nexus of several UN Sustainable Development Goals (SDGs)—food security (SDG 2), good health (SDG 3), clean water (SDG 6), economic growth (SDG 8), responsible consumption and production (SDG 12), and protection of life on land (SDG 15). It involves goal conflicts, conflicts between actors, and uncertainties regarding pesticides’ effects on human and environmental health and the economic implications of plant protection alternatives. Pesticide use causes pollution risks globally (Tang et al. 2021) and contributes to biodiversity loss (Sánchez-Bayo and Wyckhuys 2019). While rising public attention and initiatives like the EU’s Farm-to-Fork Strategy create opportunities for sustainable transformation (Schebesta and Candel 2020), recent policy responses to growing food insecurity might result in increased pesticide use (Strange et al. 2022).

We developed our argument through abduction, i.e., the creative construction or refinement of theory based on new empirical insights (Timmermans and Tavory 2012). Our interdisciplinary research team comprises members with theoretical knowledge of evidence use for sustainability and members with multifaceted empirical knowledge of pesticide governance and use. In a year-long, iterative knowledge integration process consisting of integration by a leader and common group learning (Hoffmann et al. 2017), we brought together theoretical propositions about actors’ evidence use and empirical insights from pesticide decision-making. The former were inspired by behavioral logics related to evidence supply and demand (McNie 2007; Dewulf et al. 2020) and by stages of evidence use (Rickinson et al. 2021); the latter considered the need to analyze pesticide policies and practices concurrently and from multiple disciplinary angles (Möhring et al. 2020b). Working back and forth between theory and empirics, we specified a variety of barriers to evidence use for sustainability.

In this perspective article, we systematically discuss theoretical and empirical literature that we judged most relevant to stimulate scholarly debate on actors’ evidence use. First, we introduce our argument about the interaction of different actor motivations within stages of evidence use into the discourse on science for sustainability. Second, we empirically apply the argument to pesticide risk reduction and identify manifold barriers to a transformative impact of scientific evidence. Third, we reflect on the argument’s limitations. Fourth, we conclude by deriving recommendations on how to improve evidence use for tackling pesticide risks and other sustainability challenges.

## Actor motivations and stages of evidence use

Our argument builds on the premise that scientific evidence can contribute to sustainability transformations by informing policy and practice. Scientific evidence denotes the explicit interpretation of information, data, or facts generated through a formalized process and used to support or refute certain statements or arguments (Majone 1989; Raymond et al. 2010). Along pragmatist lines, we understand science to provide tentative truths and uncertainty evaluations that may change over time (Johnson and Onwuegbuzie 2004). Policies are outputs of governance arrangements in which public and private actors seek solutions to societal problems (Knill and Tosun 2020). Policies typically seek to shape practices comprising techniques, methods, and procedures in public or private service delivery, production, marketing, or consumption. Cumulative policy and practice changes represent sustainability transformations when converging around outcomes that foster human development within planetary boundaries (Patterson et al. 2017). Sustainability is achieved in the “safe and just operating space” that provides the socio-economic foundations for human development without surpassing the environmental ceiling (Dearing et al. 2014). We consider that, while not being a panacea, the use of scientific evidence can unfold transformative impact, i.e., it can help identify and select pathways into this space.

A transformative impact toward sustainable policies and practices is a main purpose of sustainability science (Caniglia et al. 2021; Tengö and Andersson 2022). Existing research in environmental governance (Haas 2004), science and technology studies (Callon 1986; Nimmo 2016), knowledge translation and utilization (Heinsch et al. 2016), and evidence-based policy-making and practice (Boaz et al. 2019) has shown that scientific evidence can inform policy and practice change. Science communication research has generated guidelines on how scientists can increase evidence use further (Rose et al. 2020). Work on actor-worlds (Callon 1986) and -scenarios (Borst et al. 2019), however, suggests that targeted actors differ in how they translate evidence into action. Knowledge translation scholars argue that key messages need to be selected for different actors and tailored to, inter alia, their needs, interests, norms, and routines (Grimshaw et al. 2012; Hoffmann et al. 2019). Others added that intense exchange between researchers and targeted actors can ensure knowledge use in policy and practice (Gredig et al. 2021). Drawing on research on environmental governance, evidence use, and knowledge for sustainability, we develop an argument that captures a broad variety of barriers to a transformative impact of evidence and reconciles existing recommendations on how to overcome them.

At the core of evidence use are actors, defined here as human individuals or organizations that have “the capacity to comprehend a given situation or reflect upon a set of circumstances and to act in order to reshape these circumstances to a greater or lesser degree” (Nimmo 2016, p. xxvi). Actors can assume the political roles of policymakers (e.g., parliamentarian) or stakeholders (e.g., Farmers’ Union) and practice roles along the value chain, from producers (e.g., farmer) to intermediaries (e.g., retailer) and consumers. Roles sometimes overlap, for instance, when individuals are both practice actors and members of political organizations. Human agency both produces and is embedded in socio-economic and political structures circumscribing the leeway and influence in decision-making.

We advocate studying how different motivations of policy and practice actors interact within stages of evidence use to identify barriers to evidence use for sustainability. We distinguish five stages of evidence use (cf. Rickinson et al. 2021) broadly understood: [1] evidence production; [2] evidence uptake; [3] influence of evidence on co-evolving policies and practices; [4] effects of evidence-informed policies and practices on sustainability outcomes; and [5] new evidence production using feedback from the evaluation of effects. We argue that evidence use in these stages can best be understood through the complementary application of three ideal-typical actor motivations. Each stylized motivation involves a distinct logic of how actors treat scientific evidence. Depending on the prevailing motivations, the main barriers to the transformative impact of evidence are rooted in its supply, demand, or mismatch of both (McNie 2007). Building on actor-centered work, our argument underlines that actors with different motivations may shape different stages of evidence use and that motivations may even vary in between stages. Recognizing this diversity will produce a comprehensive assessment of barriers to evidence use for sustainability. It can inform the choice of measures for increasing evidence use proposed in different streams of literature.

First, *truth-seeking* actors make decisions based on the best available scientific evidence. This presupposes assigning truth values to evidence and often also constructing evidence hierarchies (cf. Cairney 2016). For truth-seeking policymakers and practitioners, more and better evidence supply by scientists (i.e., push) facilitates the identification and selection of pathways toward sustainable transformation (Haas 2004; Montpetit and Lachapelle 2015). Notably, evidence helps tackle sustainability challenges by decreasing uncertainty, mapping complexity, and triggering changes in previously conflicting preferences (Haas 2004; Messerli et al. 2019).

Second, *sense-making* actors seek to integrate scientific evidence into their belief systems (Dewulf et al. 2020). For such policymakers and practitioners, the impact of science on preferences and perceived uncertainty and complexity depends on the match of the needed and supplied evidence. This match includes the resonance of scientific evidence with the actors’ individual experiential knowledge (Raymond et al. 2010) and their need for both problem-oriented (causal) and solution-oriented (actionable) knowledge (Caniglia et al. 2017; Tengö and Andersson 2022). Meaningful knowledge co-production (Norström et al. 2020), a multiple evidence base drawing on different knowledge systems (Tengö et al. 2014), and target-specific science adaptation and dissemination at windows of opportunity (Rose et al. 2020) also facilitate transformative impact.

Third, *utility-maximizing* actors strategically employ scientific evidence to pursue predefined interests. Utility varies across actors, for instance, incumbent firms might want to protect established business models, whereas civil society organizations might pursue goals congruent with the public good. Either way, strategic actors use evidence to substantiate their preferences in political conflicts and change others’ perceptions of uncertainty and complexity accordingly (Weiss 1979; Choi et al. 2005). This strategic demand for evidence (i.e., pull) shapes evidence uptake and, possibly, generation; and its effects on sustainability depend on whether these actors are interested in sustainable transformation.

While utility-maximizing, sense-making, and truth-seeking reflect distinct logics of treating evidence, an actor may be driven by varying motivations within this spectrum depending on the decision context. We propose these motivations as analytical lenses for grasping actor-related barriers to evidence use, but we will also offer some normative considerations about increasing evidence use for sustainability.

**Fig. 1:**
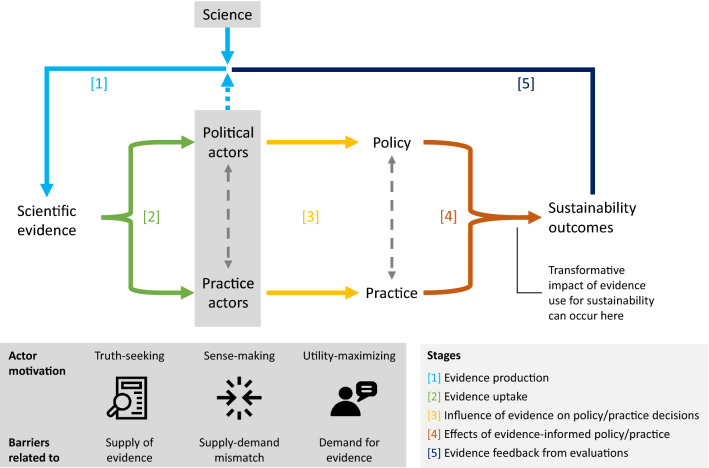
Actor motivations and stages of evidence use for sustainability. Political and practice actors and their relations influence scientific evidence production [1], determine evidence uptake [2], and shape how evidence influences decisions in the co-evolving policy and practice arenas [3]. By implementing evidence-informed policies and practices, actors can transform sustainability outcomes [4] whose evaluation may serve to generate new evidence [5]. Barriers to the transformative impact of evidence differ depending on actor motivations in each stage of evidence use. *Source* Authors; icons: first two icons made by Freepik from www.flaticon.com; third icon made by Karacis from www.flaticon.com (all subject to Flaticon license)

Systematically applying the actor motivations across stages of evidence use has several advantages. By considering all major stages, our approach is open-ended regarding where barriers to the transformative impact of evidence are located (Fig. 1). While gaps in evidence production [1] constrain truth-seekers, barriers may also emerge in other stages [2–5]. By placing actors at its center, our approach considers that uncertainty perceptions, beliefs, interests, and power relations can modulate the impact of evidence (Ingold and Gschwend 2014; Patterson et al. 2017). For instance, scientists’ insufficient evidence adaptation to *sense-makers* (Hoffmann et al. 2019) and *utility-maximizers*’ strategic evidence uptake [2] may limit this impact. Additionally, the value trade-offs that actors face regarding sustainability problems (Messerli et al. 2019; Tengö and Andersson 2022) may shape how evidence influences their decisions [3]. By covering both policy and practice, this approach assesses these and other barriers in two decision-making arenas that are interconnected through co-evolutionary dynamics (Boaz et al. 2019; Edmondson et al. 2019).

Gaining a holistic understanding of actor motivations in stages of evidence use demands interdisciplinary integration. Combining inputs from natural, health, social science, and other disciplines allows researchers to: identify a broad range of evidence on biophysical, socio-economic, and other relevant aspects of sustainability problems; trace actors’ evidence use in social, economic, and political terms; and analyze the sustainability outcomes of evidence-informed decisions in terms of socio-economic foundations and environmental ceilings. Next, we study an exemplary sustainability challenge from an interdisciplinary angle to illustrate the variety of barriers to evidence use resulting from diverse actor motivations within stages of evidence use.

## Barriers to evidence use in pesticide policy and practice

**Fig. 2:**
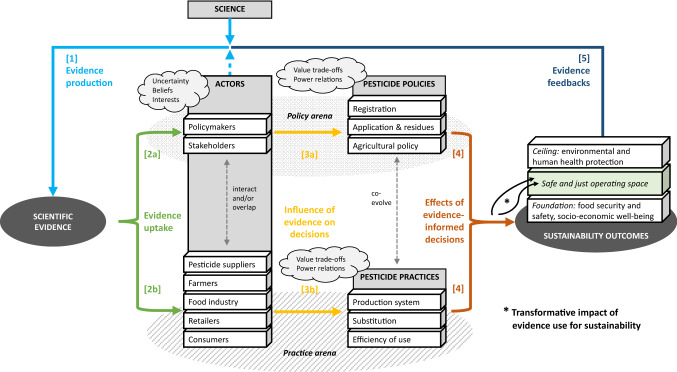
Evidence use for sustainable pesticide risk reduction. Policymakers, stakeholders, and value chain actors influence scientific evidence production regarding pesticide use, exposure, and effects [1]. Actors’ uncertainty perceptions, beliefs, and interests shape evidence uptake [2]. Power relations modulate evidence’s influence on pesticide-related policy and practice decisions characterized by the trade-offs between objectives [3]. Implementing more or less evidence-informed decisions produces sustainability outcomes [4] whose evaluation provides feedback for new evidence production [5]. Evidence use has a transformative impact when facilitating the selection of pathways into a safe and just operating space. *Source* Authors

We empirically explored evidence use for sustainability in agricultural pesticide policy and practice (Fig. 2). Pests and diseases cause yield losses of 17%–30% globally (Savary et al. 2019). A widespread strategy to control them and to ensure agricultural product quality is the application of pesticides, which entail environmental and human health risks. Studying how actor motivations within different stages of evidence use may block a transformative impact of evidence toward sustainable risk reduction of pesticides, we concentrated on:

- Scientific evidence, especially about the adverse external effects of agricultural pesticide applications on environmental and human health.
- Pesticide-related policies, including the regulation of registration, application, and residue levels of plant protection products and general agricultural policy.
- Pesticide-related practices in agriculture with risk reduction measures, such as efficiency gains in pesticide use, substitution, and system re-design (Möhring et al. 2020b).
- Sustainability outcomes, comprising socio-economic well-being (e.g., viable farms) and food security and safety without posing excessive risks for environmental and human health.

We synthesized insights from environmental and health sciences, political science, decision analysis, agricultural economics, and agronomy. Our focus was on cutting-edge research from these disciplines and fields that helps infer barriers to evidence use for sustainability. We focused on the Global North, especially Europe, where recent policy initiatives (Schebesta and Candel 2020) and zero-pesticide visions herald potential momentum for sustainable transformation. We applied the three stylized actor motivations across the five stages of evidence use to identify a variety of barriers to the transformative impact of scientific evidence (Table 1). Below, we explain for each stage the barriers resulting from the different actor motivations.

**Table 1:** Potential barriers to the transformative impact of evidence. *Source* Authors

Stage of evidence use	Actor motivations		
	Truth-seeking	Sense-making	Utility-maximizing
1. Evidence production	Evidence gaps create uncertainties	Imbalance of causal and actionable evidence	Status quo actors influence evidence production
2. Evidence uptake	Uncertainties contest need for policy/practice change	Evidence not matching actors’ prior beliefs	Actors’ interests guide evidence provision or uptake
3. Influence of evidence on policy and practice decisions	Low evidence accumulation prevents innovation	Available evidence does not match actors’ needs	Powerful status quo actors limit influence of evidence
4. Effects of evidence-informed policies and practices	Unintended effects due to siloed evidence base	Ineffectiveness due to mismatch with needs of targeted actors	Implementation deficits due to neglect of interests of key actors
5. Evidence feedback	Time lags and missing data/ tools in evaluation of effects	Scientific and experiential evaluations diverge	Status quo interests impede appropriate feedback

### Stage 1: Evidence production

Applying the three actor motivations to evidence production reveals that science’s limited transformative impact may be linked to evidence gaps, imbalances in the type of evidence produced, and the strategic shaping of evidence by status quo interests.

*Truth-seeking* actors can find ample evidence for pesticides’ adverse effects on environmental and human health. Agricultural pesticides often constitute a major ecotoxicological risk to aquatic life (Schulz et al. 2021), frequently reach critically high concentrations in pollen as food for key pollinators like honeybees (Zioga et al. 2020), and are widespread in soils (Riedo et al. 2021). Environmental science has found abundant evidence for pesticide toxicity on non-target organisms and its subsequent effects on higher biological levels, such as community structures and functions (Gunstone et al. 2021; Schulz et al. 2021). Moreover, specific pesticides have numerous human health consequences (Kim et al. 2017). Researchers have found headaches, sleep problems, and respiratory disorders as some of the acute pesticide exposure symptoms and associated chronic exposure to even low pesticide levels with neurological and mental disorders, reproductive problems, and cancer (Ohlander et al. 2020). However, assigning causality to observed correlations, especially for chronic effects, remains challenging (Ohlander et al. 2020). Little knowledge also exists about the integrated net exposures of different population subgroups (e.g., farming/non-farming and urban/rural) to specific chemicals and the contributions of different sources (e.g., occupational and residential exposure). Disentangling the quantitative contributions of single factors, such as pesticide use in multi-stressor contexts, is another key challenge (Wagner et al. 2021). Although sufficient evidence exists to justify pesticide risk reduction actions, *truth-seeking* decision-makers have to cope with evidence gaps and uncertainties.

Evidence gaps are more nuanced in the case of *sense-making* actors; for them it matters which type of evidence is needed and supplied. Many researchers recommend managing pests by promoting natural enemies, but actionable evidence for implementing such conservation biocontrol is lacking. The available evidence misfits farmers’ needs in practical decisions and fails to address conservation practitioners and policymakers that could create incentives for adoption (Chaplin-Kramer et al. 2019). Producing more causal evidence regarding pesticides’ adverse effects cannot overcome such mismatches.

Exclusively focusing on evidence gaps or mismatches neglects that some *utility-maximizing* actors try to strategically influence evidence production to accelerate, deviate, or stop transformative processes. Transformation, and the evidence supporting it, promote or threaten actors’ interests. For instance, pressured by environmental NGOs, European regulatory bodies have used scientific expertise to substantiate calls for regulatory intervention on neonicotinoids (Rimkutė 2015). In other cases, input suppliers with commercial stakes in maintaining the status quo funded research that challenged evidence about pesticides’ negative externalities (Rohr 2021).

### Stage 2: Evidence uptake

After scientific evidence production, the various motivations of policy and practice actors suggest that uncertainties related to available evidence, limited resonance of evidence with actors’ beliefs, and an interest-guided selection and interpretation of evidence may limit evidence uptake.

For *truth-seeking* decision-makers and stakeholders, uncertainty can limit evidence uptake. In the policy arena [2a] (Fig. 2), uncertainties may fuel controversy over the need for pesticide policies and further regulation. Relevant uncertainties comprise the causal inferences when multiple environmental stressors are present (Wagner et al. 2021) and the adverse human health effects of pesticide use (Ohlander et al. 2020) and reduction (e.g., farmers suffering from increased stress). However, as explained above, these uncertainties are not large enough to question pesticide risk reduction efforts. In the practice arena [2b], uncertainty-reducing evidence about pesticide effects can change the perceptions, beliefs, and preferences of farmers, the key actors in pesticide use. For example, providing toxicity information on pesticides as a nudge in the form of labels can incentivize farmers to adjust their production practices toward lower pesticide risks (Buchholz and Musshoff 2021).

Alternatively, evidence uptake may fail when scientific evidence does not match *sense-making* actors’ basic knowledge and convictions. In the policy arena [2a], many conflicts over future food systems are rooted in controversies about what knowledge is relevant and credible (Turnhout et al. 2021). Framing evidence around pressing actor-specific and societal needs can increase its perceived relevance (Rose et al. 2020). In the practice arena [2b], another important prerequisite for uptake is the evidence’s resonance with actors’ concerns and moral considerations. For instance, French farmers were willing to change their farming practices to reduce the risk of adverse effects on human and environmental health if they perceived pesticides to have an important impact on the environment (Chèze et al. 2020). This example shows how evidence regarding adverse pesticide effects can potentially support transformation.

Eventually, evidence uptake may be strategic when *utility-maximizing* actors’ interests predetermine what evidence is considered and how it is interpreted. In the policy arena [2a], for example, actors vary in their priorities regarding European ecological risk assessments of pesticides. While academics strive for assessments with higher ecological relevance, regulators favor sufficiently protective and easy-to-follow assessments, and the industry prefers more probabilistic approaches (Hunka et al. 2015). Likewise, in the practice arena [2b], interests may shape evidence communication and uptake. For example, among the extension services offering research- and knowledge-based farming advice, farmers advised by public extension services are more likely to use non-chemical preventive measures (e.g., nets) to avoid invasive species infestations, while those advised by private extension services are more likely to use synthetic insecticides (Wuepper et al. 2021). The latter practice is also problematic for farmers because it contributes to the resistance evolution of pests.

### Stage 3: Influence of evidence on policy and practice decisions

Despite its uptake, not all evidence translates into policy and practice decisions. Considering once again the different actor motivations, the potential reasons include insufficient evidence accumulation, mismatches with decision-makers’ evidence needs, and actors’ interests and power relations.

Sufficient evidence accumulation is critical for informing *truth-seekers*’ decisions. In the policy arena [3a], the scientific evidence accumulation regarding adverse pesticide effects is reflected in the emergence of several pesticide policies and programs, codes of conduct, and national action plans in European countries (Lee et al. 2019). Additionally, evidence materializing in technological innovation can be transformative: digitization facilitates novel policy designs (Ehlers et al. 2021), and advances in precision farming lead policymakers to create incentives for farmers to use them (Finger et al. 2019). In the practice arena [3b], more scientific evidence on alternative agricultural models (e.g., agroecology) could support the development of innovative plant protection solutions. Such solutions would increase the economic feasibility of phasing out widely-used but contentious pesticides, such as glyphosate (Clapp 2021a).

*Sense-makers* primarily integrate those pieces from the accumulated evidence into decision-making that match their predispositions and needs. In the policy arena [3a], an important predisposition is risk culture, which shapes the treatment of uncertain evidence. For instance, the precautionary principle facilitates policy action despite uncertainty (Metz and Ingold 2017). In the practice arena [3b], scientific evidence needs to resonate with sense-makers’ belief systems and experiential knowledge. Farmers’ decisions to reduce pesticide use are linked, among others, to: the belief that they have control over their production (Knapp et al. 2021); the knowledge of sustainable farming practices (Dessart et al. 2019); and whether other farmers also implement risk reduction measures (Bakker et al. 2021). Importantly, the decisions in both policy and practice involve value trade-offs, and to deal with them, *sense-makers* need not only problem-oriented but also solution- and preference-oriented knowledge (Box 1).

Additionally, *utility-maximizers*’ interests and power resources shape which evidence is valued in decision-making. In the policy arena [3a], the observed cross-country differences in banned pesticides (e.g., between the US and EU countries) (Donley 2019; Clapp 2021a; Rohr 2021) reflect influential actors’ interests. In many countries, farmers’ associations have long enjoyed privileged institutional access to define agricultural policy priorities, but consumer groups and retailers have now begun to challenge them (Daugbjerg and Feindt 2017). Owing to recent mergers, agrochemical companies have expanded their ability to influence policy through lobbying, framing, and structural power (Clapp 2021b). In the practice arena [3b], evidence about pesticide effects interacts with the perceived costs, benefits, risks, and other behavioral factors of decision-making (Dessart et al. 2019). Farmers’ leeway is restricted by consumer preferences and the costs of pesticide inputs, prices, and quality standards set by the food industry and retailers. Integrated pest management strategies in European maize-based cropping systems, for example, can significantly reduce pesticides’ adverse effects on human and environmental health, but lack of consumer awareness and acceptance may inhibit their adoption (Vasileiadis et al. 2013).

Box 1 Value trade-offs and scientific evidence The trade-offs inherent in sustainability problems mean that all actors’ objectives cannot be achieved simultaneously. As confirmed by studies that assessed agricultural sustainability with a multi-criteria approach, trade-offs exist between socio-economic, environmental, and other objectives (Mouron et al. 2012; Lavik et al. 2020). The extent to which agricultural stakeholders value objectives differently may also depend on how they use evidence from different scientific disciplines. Conflicting objectives impede reaching a consensus about sustainable transformation.

One way to tackle trade-offs in complex sustainability problems like pesticide risk reduction is Multi-Criteria Decision Analysis (MCDA) (Keeney and Raiffa 1976). MCDA assesses the performance of options and strategies in policy and practice using a set of objectives reflecting the actors’ aims and values. Performance assessment is informed by causal and actionable evidence on how well options achieve objectives and considers uncertainties. This assessment, based on the transparent use of scientific evidence, can generate solution-oriented knowledge and integrate preference-oriented knowledge that *sense-making* and *utility-maximizing* actors need to adequately deal with trade-offs. This knowledge combination can feed into decision support and facilitate compromise solutions (Gregory et al. 2012).

A major limitation of previous studies on pesticide management in European agriculture is that they did not elicit actors’ preferences about the trade-offs they are willing to make. An instructive example is the comparison of four management strategies, including pesticide use and innovative crop protection measures, in Norway (Lavik et al. 2020). The assessment results of the pest management strategies are determined by the equal weights assigned to the relevant objectives, which were assumed rather than elicited. Participatory MCDA that elicits the preferences of farmers, stakeholders, and policymakers can produce more sophisticated and actionable evidence for policy and practice decisions.

### Stage 4: Effects of evidence-informed policies and practices

Even decisions informed by scientific evidence may fail to produce the desired sustainability outcomes. Depending on the actor motivations, major barriers in this stage arise from a siloed evidence base or the neglected needs or interests of the actors crucial for implementation.

Evidence-informed policies and practices of *truth-seeking* actors may fail to produce the desired sustainability outcomes due to unintended effects of attempts to address complex problems through single policy instruments. Although market-based instruments, such as Denmark’s pesticide taxes, can reduce pesticide use, complementing them with other instruments (command and control, subsidies, information, and extension) can increase effectiveness (Lee et al. 2019; Pedersen et al. 2020). Instrument mixes that target different sectors and value chain actors demand cross-sectoral coordination (Wiedemann and Ingold 2021). Interdisciplinary rather than siloed evidence best informs such policy efforts and agricultural practice decisions with biological, economic, regulatory, and other parameters (Box 1).

Under *sense-making*, evidence-informed policies and practices are only effective when the needs of implementing actors are factored in. Effective pesticide policies consider the behavioral predispositions of value chain actors—e.g., that economically more risk-averse farmers use more toxic pesticides (Möhring et al. 2020a). Furthermore, sustainable and commercially viable plant protection practices emerge from an interplay of causal and actionable scientific evidence, practitioners’ experiential knowledge, and agricultural policy. One such interplay is exemplified by the transformation of Swiss apple production toward integrated pest management and organic production (Box 2).

Similarly, evidence-informed policies may lack effectiveness when the interests of *utility-maximizing* actors that shape implementation are neglected. A lack of support from key actors may render it difficult to implement and sustain policies; and policies fail to transform pesticide practices when on-the-ground diversity and complexities are ignored. For instance, the heterogeneity of farmers’ preferences and goals creates a need for multiple policy instruments (Pedersen et al. 2020).

Box 2: Evidence and the transformation of plant protection in Swiss apple production Swiss apple production’s history is closely linked to evidence regarding pesticide effects and alternative plant protection methods. Intensive synthetic pesticide use in agriculture began with the development of DDT (dichlorodiphenyltrichloroethane) in the 1940s. From the late 1940s onwards, Swiss apple farmers started increasingly using the insecticide classes organochlorines (e.g., DDT), organophosphates (e.g., parathion), and carbamates and fungicide classes like dithiocarbamates (e.g., mancozeb) and phthalimides (e.g., captan).

Public doubts regarding intensive pesticide use arose globally in the early 1960s. Rachel Carson’s *Silent Spring* (1962) collected problem-oriented evidence on adverse pesticide impacts and solution-oriented evidence on alternatives. This evidence uptake increased public awareness and led to a DDT ban in agriculture. In the 1960s and 1970s, several ‘modern’ pesticides became available, including new fungicides (benzimidazole, metalaxyl, and triazoles) and insecticides (pirimicarb, pyrethroids, and neonicotinoids). Their intensive use transformed apple production into highly productive orchards but adversely affected other sustainability outcomes (e.g., benzimidazoles triggered fungicide resistance and spider mite multiplication).

The 1960s and 1970s saw an increasingly unsustainable Swiss apple production but also innovative farmers and advisors who adopted the novel concept of Integrated Pest Management (IPM), including the idea of applying pesticides only when pests surpass economically damaging thresholds. Farmers, researchers, and advisors jointly began to develop IPM systematically for Swiss apple production. Regional farmer groups fostered knowledge transfer and the development of a premium brand, and one of the two major Swiss retailers, Migros, promoted IPM practices with a distinct label (‘M-Sano’). Additionally, organic farming organizations disseminated evidence-based guidance on biological and biotechnical methods (e.g., pheromones).

In the 1980s and 1990s, with the emergence of more problem- and solution-oriented evidence, the transformation of Swiss apple production accelerated. Researchers recognized the need to quantify pesticide impacts on non-target organisms, and the Swiss legislator refined the data requirements for pesticide registration and further restricted chemicals. Commercially, the premium brand for IPM (‘IP’) was launched, and the other major Swiss retailer, Coop, initiated its organic brand. In farming, innovative non-chemical alternatives for pest and disease control (e.g., granulosis viruses against codling moth) allowed for restricting insecticide and fungicide treatments. Elements promoting functional biodiversity (e.g., perennial flower strips) further reduced dependency on insect pest control.

In the 2000s and 2010s, the apple production practices continued to become more sustainable. By providing actionable evidence for timely pesticide application, the weather-based Decision Support Systems (DSS) revolutionized scab control, and new scab-resistant apple varieties were introduced to compensate for resistance breakdowns. Recently, retailers have developed new private standards to promote the production of apples with only low levels of pesticide residues. In organic apple production, post-infection treatments with non-synthetic chemicals like lime sulfur or potassium bicarbonate against scab infections have been an evidence-based breakthrough.

Today, Swiss apple production complies with IPM standards or certified organic production. Despite such progress, apples still require intensive pest and disease control and the public remains concerned about pesticide impacts. Continuous feedback, production, and uptake of evidence are needed to advance the co-evolution of sustainable policies and practices addressing new pests and diseases.

### Stage 5: Evidence feedback

Weak feedback from sustainability outcomes to renewed evidence production can also hinder transformation. A sensitivity for different actor motivations reveals that this may be due to time lags and missing data, diverging results of scientific and experiential evaluations, and strategic impediments by status quo interests.

*Truth-seeking* actors’ efforts to improve pesticide policies and practices using evidence feedback on previous sustainability effects are hampered by gaps in monitoring and evaluation. An example is the environmental effects for which model-based proxies (Schulz et al. 2021) have to compensate for the lack of consistent long-term data linking meaningful quantitative metrics on pesticide use, exposure, and ecological effects. An even more instructive example is human health evaluations, which often lag decades behind regulation and farmers’ practices and exposure (Ohlander et al. 2020). To provide conclusive data on health effects, especially chronic ones, long-term cohort studies are needed. Such epidemiological evidence can only be gathered after pesticides’ market introduction, and it may take several decades to observe effects (e.g., cancer). The collection of personal exposure data (e.g., urine or blood samples) is costly, logistically and ethically challenging, or became possible only recently (e.g., passive sampling via wristbands).

*Sense-makers* interpret evidence feedback through the lens of their experiences and beliefs. For instance, from a long-term Swiss pilot project that sought to improve water quality through the voluntary adoption of good pesticide practices, its key actors drew different intermediate conclusions. While the farmers and authorities involved in the implementation interpreted the results as successful, scientists could not establish a clear causal link between the adopted measures and monitored water quality (Daouk et al. 2019). Additionally, long timelines for regulatory revisions may prevent quick incorporation of evidence feedback into policy (Topping et al. 2020).

The status quo interests of *utility-maximizing* policy and practice actors may prevent the available evidence from monitoring and evaluations from being fed back into decision-making. Despite pesticide registration being in place for decades, significant feedback deficits exist since monitoring is hardly considered during registration (Topping et al. 2020; Siviter et al. 2021). Furthermore, the registration and guidelines for water quality assessment consider only single compounds or products, neglecting well-established mixture toxicity and the co-occurrence of numerous pesticides in the environment.

## Critical reflections

The empirical illustration of our argument demonstrates that filling evidence gaps on adverse pesticide effects alone is unlikely to trigger sustainable transformation. The reason is that diverse actor motivations play out within different stages of evidence use. Not all actors are technocratic *truth-seekers* that are constrained by evidence gaps. Other serious barriers to a transformative impact of evidence are mismatches between *sense-makers*’ evidence needs and the available evidence and *utility-maximizers’* strategic evidence use to protect status quo interests.

Several points of critique could be raised against our argument. To begin with, our abductive approach may be criticized for mixing empirical observations and theory development. We agree that the pesticide case presented here shall not be taken as theory confirmation but rather as a source of empirical insights that inspired theory development. Accordingly, our propositions about the roots of barriers to evidence use in different actor motivations and stages remain to be tested in other empirical contexts.

Other critiques may concern our model’s conceptual foundations. Actor-network theorists may find our actor concept too restrictive as they also consider how nonhuman entities, such as materials, technologies, organisms, and ecology “act” on humans (Nimmo 2016). For instance, we have not discussed how changing pest pressure influences evidence production and uptake by input suppliers and farmers. Furthermore, and similar to the policy cycle concept (Cairney 2016), our depiction of unidirectional, sequential stages of evidence use risks oversimplification. Real-world evidence use may be messy, moving back and forth between stages, as shown in Box 2. Likewise, the three actor motivations we distinguished paint over many shades. Shades deserving further exploration include *truth-seekers’* preoccupation with the legitimacy of different evidence types and sources (Dewulf et al. 2020), the psycho-cultural underpinnings of the meanings that render certain aspects of reality pertinent to *sense-makers* (Salvatore et al. 2019), and the extent to which even strategic *utility-maximizers* partially adapt their goals in light of new knowledge (Dewulf et al. 2020). Recognizing these limitations, our model’s value lies in its use as a simple heuristic that can grasp various barriers to evidence use arising from human agency and can stimulate scholarly debate.

Finally, our assumption that evidence use can contribute to sustainable transformation may be criticized for an implicit truth-seeking focus. We followed pragmatism’s middle position in considering that scientific evidence provides tentative answers of practical value on the long-term move toward larger truths (Johnson and Onwuegbuzie 2004). We are also sympathetic to the more realist idea that a proposition’s (provisional) truth presupposes empirical support and consistency with relevant background knowledge (Bunge 2014). Recognizing, however, that philosophy of science debates remain controversial, we believe that our framework is sufficiently flexible to accommodate or be adapted to other stances scholars might take. In this context, also the normative question of whether *truth-seekers* are “better” than *sense-makers* and *utility-maximizers* arises. While considering all three motivations legitimate, we posit that conditions for enhancing the transformative impact of evidence can be created, as discussed below.

## Conclusion and recommendations

To study the use of scientific evidence for sustainability, we outlined three complementary actor motivations within five stages of evidence use. We argued that paying attention to this diversity helps capture the manifold barriers to the transformative impact of evidence. We empirically illustrate such barriers in the policy and practice of reducing environmental and human health risks of agricultural pesticides. The observed variety of barriers implies that no one-size-fits-all solution for enhancing evidence use exists. Instead, actors serving public interests, including policymakers, public administrations, and researchers, can adopt context-specific research–policy–practice measures to increase evidence use for sustainability.

To confront sustainability challenges like pesticide risk reduction, researchers initially can identify the dominant barriers to the transformative impact of evidence as well as bright spots of science-informed policies and practices (Cvitanovic and Hobday 2018). The social sciences in collaboration with other disciplines can map interacting motivations of influential policy and practice actors in all stages of evidence use, taking into account varying contextual conditions (e.g., problem structure and regulatory system). Empirical findings on dominant barriers to evidence use and on success cases of overcoming them will suggest the extent to which the following three reform packages could be applied:

First, if the decision-makers are primarily *truth-seeking* but constrained by evidence gaps, the evidence supply should be enhanced. The collection and accumulation of evidence can be improved horizontally across sectors (e.g., via interdisciplinary integration), vertically between levels (e.g., via connecting global assessments and local knowledge bases), and in time (e.g., via dynamic evidence syntheses). Incorporating evidence feedback into decision-making can be expanded through more financial and human resources for dynamic evidence summaries and transparent evaluation programs with clearly defined purposes and multi-directional information flows (Topping et al. 2020). Where research cannot close evidence gaps, clear principles and guidelines for decision-making under uncertainty can ensure transparency.

Second, if the influential actors behave like *sense-makers*, the match between evidence supply and demand should be increased. Knowledge translation literature suggests that, to this end, transdisciplinary expertise for knowledge co-creation and related interactions between scientists, policymakers, and practitioners can be promoted (Hoffmann et al. 2019; Norström et al. 2020). Boundary organizations can strengthen their integrative capacity in brokering evidence for policy or practice (McNie 2007) and academia can create favorable conditions for solution-oriented research that actors need (Lang and Wiek 2022). Participatory evaluation research that integrates actors’ experiential knowledge and monitoring data can prevent mismatches in evidence feedback.

Third, if strategic *utility-maximizing* actors dominate, safeguards against evidence misuse by vested interests should be introduced (Rohr 2021). A crucial safeguard can be greater transparency regarding evidence demand and use. Institutional arrangements for public data transparency can prevent data monopolies that allow using or holding back evidence selectively. As per Box 1, participatory multi-criteria decision analysis makes elicited stakeholder preferences transparent, for instance, in the form of the weights attributed to conflicting objectives (Keeney and Raiffa 1976; Gregory et al. 2012). Additionally, a requirement to attach evidence documentation to legislative and administrative acts can render evidence use in policy-making more traceable.

These recommendations likely apply beyond pesticide risk reduction in the Global North. Being cognizant of contextual differences (e.g., climate, indigenous knowledge), they may also inform strategies for reducing major pesticide risks in the Global South (Tang et al. 2021). Many barriers to evidence use observed in pesticide risk reduction will also be relevant to other sustainability challenges characterized by complexity, conflict, and uncertainty. We encourage others to investigate systematically how actor motivations interact in the generation, flow, and use of scientific evidence as a deep leverage point for sustainability transformations (Abson et al. 2017). Such studies will help refine our tentative recommendations for tapping the full transformative potential of science in pursuing human development within planetary boundaries.
